# Supplementary material for: Patterns of Intron Gain and Loss in Fungi
Source: PLoS Biol. 2004 Nov 30;2(12):e422. doi: 10.1371/journal.pbio.0020422 (PMC532390; doi:10.1371/journal.pbio.0020422)
Supplement: Table S1 — Also available at http://genes.mit.edu/NielsenEtAl/. (4.3 MB ZIP). [file pbio.0020422.st001.zip › NielsenEtAl/html/905.html]

AN0312.1.NCU09247.1.MG01654.1.FG07086.1


```
 CLUSTAL W (1.82) Multiple Sequence Alignments - Introns Inserted


Sequence 1: MG01654.1	692 aa
Sequence 2: FG07086.1	688 aa
Sequence 3: NCU09247.1	716 aa
Sequence 4: AN0312.1	666 aa
Alignment Length: 732 aa
Number Identitical Residues: 132 aa
Alignment Score (without introns) 9684


MG01654.1 	MTTSATRPALQGLASSASSLPPRAPLGKKPILRTPQSLSVSTKRKRSDDPDDRLESSPTK
NCU09247.1	MTTLTPVATRSQATPLTSSAIKSILRKPTTVLGRRSREEDGES------SDGLPGSSPAK
FG07086.1 	MRPFTPTTRKLSGPAAKSSTPVRSILKPASVLGRRKAEDAGLS-----DPIDAPES-PTK
AN0312.1  	------MEEMDLSLSPSSSIASDADGQPSPRKKTRVQFDEDVE-----------------
          	              .  **          .        . . .                 

MG01654.1 	RLKHVTFDLESN-MTVDIGMRTLEETKREVRSALEDRVRG-ETEGYENLKAIFQNDRQRY
NCU09247.1	KRKTVMFNDKLN-MIKEFGGKTLDDAKREVKQALEGHARG-DDEDYDGLKEIFCPSPKRT
FG07086.1 	RRKVLFDDIRNVTVTYEVGKRTMDEVKLEVRTALEEHLRG-NDGQYDTLKELFATDKQRY
AN0312.1  	-MREISYNAETQGGSQWTTDKSAAVVREEVRRAIQRHVSGGDSEAYDRVKEVFSINP-RR
          	  : :  : .          ::   .: **: *:: :  *.:   *: :* :*  .  * 

MG01654.1 	LPPIVGDDEDCLKPQELIVYVVALSTCAPML-DRSCSDLIKIVTKCSWLGRDDGFCKAYT
NCU09247.1	IAE--EEDADAPQPQDLQVYVVALTSHAPLL-DRSCSGLIRAVLNCTWLDRDDVFARAYI
FG07086.1 	LPPVIGEDDDTLKPHELQVYVVALTGCVPILKDKACNGLVKTILNCSWLGRDDAFVKVFT
AN0312.1  	LDANGMPPSNVPTHTTLKHHLMGLLSNVAAL-DRSCNGLVQAVLDSEWLGRDESYIKLYI
          	:        :      *  :::.*   .. * *::*..*:: : .. **.**: : : : 

MG01654.1 	QFLAALISAQGTTLELVLAMIVEKFKDYRES-AWSVPDFPKVSFDTAQDRLHGGLRYLLR
NCU09247.1	QLLAALS-AKVYFRQQALSMMVDKFTETRTSTVEVVPGFPPVDSETKKERLHLGLKYLLE
FG07086.1 	HFLAALVSAQGSYLSPVLTMMVGKFTDSRSD-VWSVPDFPEVNLETMRDRLHRTLQYLLQ
AN0312.1  	RFLGNLAAAQGSYLGSVLKMLANYMGELPRG-TGRLPGYPHVHPAEAYTRVHMAMRHVLQ
          	::*. * :*:      .* *:.  : :   . .  :*.:* *       *:*  ::::*.

MG01654.1 	LFPAARPVLCSLLAKQFPYLEESKKVHTTYVKNLLLLRKYAPDLGQDIMELIVDRLVKID
NCU09247.1	LYADARPMILDIVTHNFPHTEESKTRHMEYIDHLLRLKVDKPDLERDIMELILSRLVKLD
FG07086.1 	MFPSALPVLECLLGSKFPYQDGSKRVHMSYVKNLLRVKDYLPDLRDEVFDLILNRVVKID
AN0312.1  	LVPSGSGSLSQIISSQFPFDTDSAKANIAYTQNLVKVIRYAPELQADILALITEKLVKID
          	: . .   :  ::  :**.   *   :  * .:*: :    *:*  ::: ** .::**:*

MG01654.1 	VQVQFDLDDMDDDLTAAIVMHLGSAREKDDLD-IDSDESDGESIASDDSDFDAEATTIET
NCU09247.1	VEMTLDLENDDDETTRAVLRQLEASNAKSDDE-DDDAMSDADSDDEDEEESDEQTRRILL
FG07086.1 	SQMQVDLEDGDDEITAAVMYALEKQVFTGDWEENDDDDSDTESVDSDDPDYDTEAAKTKS
AN0312.1  	VQIQVDMEDLEDEEGEEVLHAISPEAVMFAEDLDDEDDDDNASVVSDES-VEPESRRLKT
          	 :: .*::: :*:    ::  :         :  *.  .*  *  .*:   : ::     

MG01654.1 	GSTTIQKLDAIMDMLFEVYKPSFENPTSDEAKEVLGDLISDFVNLVLPTYKSRHTQFLVF
NCU09247.1	LKSKLETMDAILDLLFSIYDPVFADPDSPEALATFEDLLTDFVNVILPHLKSRHTQYLLF
FG07086.1 	ITDNVSKMDAILDTLFEVYTPHFDNPGSDVAFDMFTILLREFEYMVLPTYKSRHTQFLIF
AN0312.1  	IKENILKLDGMIDTLFEYYAPPFTSGTLDDKENALDLLLSHFSTIILPTYRSRHSQFLLF
          	 . .: .:*.::* **. * * * .         :  *: .*  ::**  :***:*:*:*

MG01654.1 	HFSQYSPELMDLFVGTLFNMAFESNRPMALRQSATAYLASYVARGAAVPAEIVRNVVELL
NCU09247.1	KFAMKSNRLMEMFIGTLFNIAFQSTRAPVVKQAAVAYLASFTARGARVQSDQVQLIAQTF
FG07086.1 	HFAQMHDRLTDAFCGQLITTAFKSNTANSTKQAAASYLASFVARGARLPGSLIRSIFGLL
AN0312.1  	HFSQSSPILVDRFAATCVQLIFNKAQPAILRQSAAAYLASFVARGAHISSEVVRDVFDLL
          	:*:     * : * .  .   *:.  .   :*:*.:****:.**** : .. :: :   :

MG01654.1 	CHHVDVQMRNHADGCRGPDLRRYKHLYCLVQALLYIFCFRWRELVAW--APELVDPSEKS
NCU09247.1	LDYIDHYRNTHTS-CRGPDIRRYGQYYAYFQGLLYIFCFRWRDLIDRDEVPASFDWDDPA
FG07086.1 	LHHLDIYRKKYEPLCRGPDLRRFHPYYSLVQATLYIFCFRWQDLIVV--APESADPEDPA
AN0312.1  	GTHLNNLRLDYEPSCRGPDLRRFGPFYSTAQALLYIFCFRWRDLTTA--AIEGDTPEQID
          	  :::     :   *****:**:   *.  *. ********::*     .      .:  

MG01654.1 	SYLGHDLEWIPGFKESMSRIIYSKFN~PLRVCSPGIVHEFADHARLLRFMYVFPLLESNK
NCU09247.1	SFLGQDLPWMKGLRQRLQVNIASKLN0PLKCCSPIIVDEFARLSHHLRLIYIYPQIEKNK
FG07086.1 	SYIGQEMEWMGTCRRDLSAQIFGKLN~PLKVCAPSIVEEFARIAHRLNFMYIYPLIESNK
AN0312.1  	ELEPEDITFPPSIRDVLHKAILSKLN~PLKVCSPAIVSQFARMSQHLNFIYVFTILETNK
          	.   .:: :    :  :   * .*:* **: *:* ** :**  :: *.::*::. :*.**

MG01654.1 	RIHLSQFASGDYSTGG--ALRDTCYDPYDESGLQLEAYFPFDPYQLPTSKRWIQDD--YV
NCU09247.1	TIHLSQFFTSSYATGG--ALRDSGFEFDHENWTHIEACFPFDPFQLPVAKRWLDLDKNYV
FG07086.1 	RIRLSQYLTATYSTGG--ALRDAGYDMQDESFHQLDPYFPFDPYQLPISKRWLEND--YV
AN0312.1  	RLRMSTYRNIAALSDPRYSHVERETRAGDDLGYQLDAYFPFDPYQLPRSRRWLEGD--YV
          	 :::* : .    :.   :  :      .:   :::. *****:*** ::**:: *  **

MG01654.1 	RWETLTGIGLG-DDDLDDASIDD---------------------NLDDDGNDYEEEDTAT
NCU09247.1	EWRSIAMLEKGGDDDSDSEDDDDDDVSEDGMGDIGDASDYESDEEVEAGGVEHEDDESTD
FG07086.1 	EWKSITGLNAE-DEDSDDADDED---------------------ELDEE--EFEDKTSTD
AN0312.1  	EWRGIPGLDDR-DDDDDSDDLEE----------------------LSDDEDDLSDGTETD
          	.*. :. :    *:* *. . ::                      :.    : .:   : 

MG01654.1 	DDGKDD-------
NCU09247.1	EERDGYHGSFRRI
FG07086.1 	SDGDDYE------
AN0312.1  	HE-----------
          	 :
```
